# Supplementary material for: Potato (Solanum tuberosum L.) tuber-root modeling method based on physical properties
Source: PLoS One. 2020 Sep 17;15(9):e0239093. doi: 10.1371/journal.pone.0239093 (PMC7498077; doi:10.1371/journal.pone.0239093)
Supplement: S1 Table — (DOCX) [file pone.0239093.s001.docx]

Parameters table of tuber-root model for three varieties of potatoes

| **Root or tuber types** | **Parameters** | **Zaodabai** | **Helanshiwu** | **Fujin** |
| --- | --- | --- | --- | --- |
| **Seed potato** | Length（mm） | *LS_Z_* ～*N*（13.24，3.70^2^） | *LS_H_* ～*N*（15.24，4.89^2^） | *LS_F_* ～*N*（13.70，5.13^2^） |
|  | Width（mm） | *WS_Z_* =0.78×*LS_Z_* + 0.39 | *WS_H_* =0.81×*LS_H_* + 0.14 | *WS_F_* =0.75×*LS_F_* + 0.66 |
|  | Height（mm） | *HS_Z_* =0.51×*LS_Z_* +2.06 | *HS_H_* =0.66×*LS_H_* + 0.80 | *HS_F_* =0.23×*LS_F_* + 4.08 |
| **Underground stem** | Initial radius（mm） | *RD_Z_* ～*N*（4.02，0.98^2^） | *RD_H_* ～*N*（5.91，1.06^2^） | *RD_F_* ～*N*（4.23，1.15^2^） |
|  | Radius reduction factor | *rD_Z_* ～*N*（0.028，0.006^2^） | *rD_H_* ～*N*（0.029，0.007^2^） | *rD_F_* ～*N*（0.018，0.007^2^） |
|  | Length（mm） | *HD_Z_* ～*N*（86.37，14.18^2^） | *HD_H_* ～*N*（88.19，16.45^2^） | *HD_F_* ～*N*（112.74，16.38^2^） |
| **Seminal root** | Initial axial angle（°） | *AZ_Z_* ～*N*（142.52，16.50^2^） | *AZ_H_* ～*N*（137.90，16.92^2^） | *AZ_F_* ～*N*（146.58，14.85^2^） |
|  | Length（mm） | *LZ_Z_* ～*N*（126.60，51.31^2^） | *LZ_H_* ～*N*（174.78，48.72^2^） | *LZ_F_* ～*N*（113.32，41.03^2^） |
|  | Root depth（mm） | *DZ_Z_* ～*N*（72.48，35.60^2^） | *DZ_H_* ～*N*（109.80，43.20^2^） | *DZ_F_* ～*N*（73.50，33.27^2^） |
|  | Initial radius（mm） | *RZ_Z_* ～*U*(0.25,0.5), p=0.14  *RZ_Z_* ～*U*(0.5,0.75), p=0.52  *RZ_Z_* ～*U*(0.75,1.0), p=0.14  *RZ_Z_* ～*U*(1.0,1.25), p=0.16  *RZ_Z_* ～*U*(1.25,1.5), p=0.02  *RZ_Z_* ～*U*(1.5,1.75), p=0.02 | *RZ_H_* ～*U*(0.45,0.65), p=0.22  *RZ_H_* ～*U*(0.65,0.85), p=0.16  *RZ_H_* ～*U*(0.85,1.05), p=0.32  *RZ_H_* ～*U*(1.05,1.25), p=0.10  *RZ_H_* ～*U*(1.25,1.45), p=0.06  *RZ_H_* ～*U*(1.55,1.70), p=0.14 | *RZ_F_* ～*U*(0.2,0.4), p=0.10  *RZ_F_* ～*U*(0.4,0.6), p=0.42  *RZ_F_* ～*U*(0.6,0.8), p=0.22  *RZ_F_* ～*U*(0.8,1.0), p=0.10  *RZ_F_* ～*U*(1.0,1.2), p=0.08  *RZ_F_* ～*U*(1.2,1.4), p=0.08 |
|  | Radius reduction factor | *rZ_Z_* ～*U*（-0.005，-0.002） | *rZ_H_* ～*U*（-0.005，-0.002） | *rZ_F_* ～*U*（-0.005，-0.002） |
|  | Axial deviation Angle（°） | *aZ_z_* ～*N*（0.67，0.26^2^） | *aZ_H_* ～*N*（0.6，0.26^2^） | *aZ_F_* ～*N*（0.8，0.3^2^） |
|  | Radial deflection Angle（°） | *jZ_Z_* ～*N*（0.05，0.03^2^） | *jZ_H_* ～*N*（0.05，0.03^2^） | *jZ_F_* ～*N*（0.05，0.03^2^） |
|  | Number | *NZ_Z_* ～*U*（14,18） | *NZ_H_* ～*U*（14,18） | *NZ_F_* ～*U*（14,18） |
| **Creeping stem** | Initial axial angle（°） | *AJ_Z_* ～*U*(30,50), p=0.06  *AJ_Z_* ～*U*(50,70), p=0.16  *AJ_Z_* ～*U*(70,90), p=0.38  *AJ_Z_* ～*U*(90,110), p=0.28  *AJ_Z_* ～*U*(110,130),p=0.12 | *AJ_H_* ～*U*(30,50), p=0.04  *AJ_H_* ～*U*(50,70), p=0.28  *AJ_H_* ～*U*(70,90), p=0.40  *AJ_H_* ～*U*(90,110), p=0.18  *AJ_H_* ～*U*(110,130),p=0.10 | *AJ_F_* ～*U*(30,50), p=0.04  *AJ_F_* ～*U*(50,70), p=0.18  *AJ_F_* ～*U*(70,90), p=0.44  *AJ_F_* ～*U*(90,110), p=0.20  *AJ_F_* ～*U*(110,130),p=0.14 |
|  | $\frac{\text{ Stem node height}}{\text{Underground stem height}}$（%） | *PJ_Z_* ～*U*(8,20), p=0.42  *PJ_Z_* ～*U*(20,35), p=0.18  *PJ_Z_* ～*U*(35,50), p=0.20  *PJ_Z_* ～*U*(50,65), p=0.12  *PJ_Z_* ～*U*(65,80), p=0.04  *PJ_Z_* ～*U*(80,95), p=0.04 | *PJ_H_* ～*U*(8,20), p=0.36  *PJ_H_* ～*U*(20,35), p=0.28  *PJ_H_* ～*U*(35,50), p=0.16  *PJ_H_* ～*U*(50,65), p=0.12  *PJ_H_* ～*U*(65,80), p=0.08  *PJ_H_* ～*U*(80,95), p=0.00 | *PJ_F_* ～*U*(5,15), p=0.30  *PJ_F_* ～*U*(15,25), p=0.32  *PJ_F_* ～*U*(25,35), p=0.16  *PJ_F_* ～*U*(35,45), p=0.14  *PJ_F_* ～*U*(45,55), p=0.02  *PJ_F_* ～*U*(55,65), p=0.06 |
|  | Length（mm） | *LJ_Z_* ～*N*（39.56，9.87^2^） | *LJ_H_* ～*N*（95.42，56.60^2^） | *LJ_F_* ～*N*（62.07，28.92^2^） |
|  | Root depth（mm） | *DJ_Z_* ～*N*（49.76，17.85^2^） | *DJ_H_* ～*N*（73.30，28.74^2^） | *DJ_F_* ～*N*（87.78，17.36^2^） |
|  | Initial radius（mm） | *RJ_Z_* ～*U*(0.9,1.1), p=0.08  *RJ_Z_* ～*U*(1.1,1.3), p=0.24  *RJ_Z_* ～*U*(1.3,1.5), p=0.32  *RJ_Z_* ～*U*(1.5,1.7), p=0.14  *RJ_Z_* ～*U*(1.7,1.9), p=0.12  *RJ_Z_* ～*U*(1.9,2.1), p=0.10 | *RJ_H_* ～*U*(0.7,1.05), p=0.06  *RJ_H_* ～*U*(1.05,1.40), p=0.10  *RJ_H_* ～*U*(1.40,1.75), p=0.32  *RJ_H_* ～*U*(1.75,2.10), p=0.40  *RJ_H_* ～*U*(2.10,2.45), p=0.10  *RJ_H_* ～*U*(2.45,2.80), p=0.02 | *RJ_F_* ～*U*(0.6,0.85), p=0.10  *RJ_F_* ～*U*(0.85,1.10), p=0.32  *RJ_F_* ～*U*(1.10,1.35), p=0.28  *RJ_F_* ～*U*(1.35,1.60), p=0.12  *RJ_F_* ～*U*(1.60,1.85), p=0.10  *RJ_F_* ～*U*(1.85,2.10), p=0.08 |
|  | Radius expansion coefficient | *rJ_Z_* ～*U*（0.13，0.15） | *rJ_H_* ～*U*（0.08，0.10） | *rJ_F_* ～*U*（0.08，0.14） |
|  | Axial deviation Angle（°） | *aJ_Z_* ～*U*(0.9,1.2), p=0.04  *aJ_Z_* ～*U*(1.2,1.5), p=0.08  *aJ_Z_* ～*U*(1.5,1.8), p=0.12  *aJ_Z_* ～*U*(1.8,2.1), p=0.36  *aJ_Z_* ～*U*(2.1,2.4), p=0.30  *aJ_Z_* ～*U*(2.4,2.7), p=0.10 | *aJ_H_* ～*U*(0.15,0.5), p=0.32  *aJ_H_* ～*U*(0.5,0.85), p=0.16  *aJ_H_* ～*U*(0.85,1.20), p=0.06  *aJ_H_* ～*U*(1.20,1.55), p=0.06  *aJ_H_* ～*U*(1.55,1.90), p=0.16  *aJ_H_* ～*U*(1.90,2.25), p=0.24 | *aJ_F_* ～*U*(0.5,0.8), p=0.04  *aJ_F_* ～*U*(0.8,1.1), p=0.10  *aJ _F_* ～*U*(1.1,1.4), p=0.10  *aJ_F_* ～*U*(1.4,1.7), p=0.14  *aJ_F_* ～*U*(1.7,2.0), p=0.26  *aJ_F_* ～*U*(2.0,2.3), p=0.36 |
|  | Radial deflection Angle（°） | *jJ_Z_* ～*N*（0.04，0.03^2^） | *jJ_H_* ～*N*（0.04，0.03^2^） | *jJ_F_* ～*N*（0.04，0.03^2^） |
|  | Number | *NJ_Z_* ～*U*（4，6） | *NJ_H_* ～*U*（5，7） | *NJ_F_* ～*U*（7，9） |
| **Creeping root** | Initial axial angle（°） | *AP_Z_* ～*U*(70,85), p=0.12  *AP_Z_* ～*U*(85,100), p=0.36  *AP_Z_* ～*U*(110,115), p=0.14  *AP_Z_* ～*U*(115,130), p=0.16  *AP_Z_* ～*U*(130,145), p=0.16  *AP_Z_* ～*U*(145,160), p=0.06 | *AP_H_* ～*U*(70,85), p=0.20  *AP_H_* ～*U*(85,100), p=0.28  *AP_H_* ～*U*(110,115), p=0.32  *AP_H_* ～*U*(115,130), p=0.16  *AP_H_* ～*U*(130,145), p=0.00  *AP_H_* ～*U*(145,160), p=0.04 | *AP_F_* ～*U*(70,85), p=0.18  *AP_F_* ～*U*(85,100), p=0.20  *AP_F_* ～*U*(110,115), p=0.24  *AP_F_* ～*U*(115,130), p=0.28  *AP_F_* ～*U*(130,145), p=0.10  *AP_F_* ～*U*(145,160), p=0.00 |
|  | $\frac{\text{ Root node height}}{\text{Underground stem height}}$（%） | *PP_Z_* ～*U*(8,20), p=0.32  *PP_Z_* ～*U*(20,35), p=0.22  *PP_Z_* ～*U*(35,50), p=0.26  *PP_Z_* ～*U*(50,65), p=0.02  *PP_Z_* ～*U*(65,80), p=0.06  *PP_Z_* ～*U*(80,95), p=0.12 | *PP_H_* ～*U*(8,20), p=0.40  *PP_H_* ～*U*(20,35), p=0.16  *PP_H_* ～*U*(35,50), p=0.22  *PP_H_* ～*U*(50,65), p=0.08  *PP_H_* ～*U*(65,80), p=0.14  *PP_H_* ～*U*(80,95), p=0.00 | *PP_F_* ～*U*(8,20), p=0.48  *PP_F_* ～*U*(20,35), p=0.28  *PP_F_* ～*U*(35,50), p=0.10  *PP_F_* ～*U*(50,65), p=0.10  *PP_F_* ～*U*(65,80), p=0.04  *PP_F_* ～*U*(80,95), p=0.00 |
|  | Length（mm） | *LP_Z_* ～*N*（182.80，76.86^2^） | *LP_H_* ～*N*（221.57，84.45^2^） | *LP_F_* ～*N*（159.33，68.22^2^） |
|  | Root depth（mm） | *DP_Z_* ～*N*（99.12，36.83^2^） | *DP_H_* ～*N*（161.68，56.31^2^） | *DP_F_* ～*N*（151.24，51.67^2^） |
|  | Initial radius（mm） | *RP_Z_* ～*U*(0.2,0.4), p=0.14  *RP_Z_* ～*U*(0.4,0.6), p=0.38  *RP_Z_* ～*U*(0.6,0.8), p=0.28  *RP_Z_* ～*U*(0.8,1.0), p=0.06  *RP_Z_* ～*U*(1.0,1.2), p=0.10  *RP_Z_* ～*U*(1.2,1.3), p=0.04 | *RP_H_* ～*U*(0.2,0.4), p=0.14  *RP_H_* ～*U*(0.4,0.6), p=0.34  *RP_H_* ～*U*(0.6,0.8), p=0.28  *RP_H_* ～*U*(0.8,1.0), p=0.14  *RP_H_* ～*U*(1.0,1.2), p=0.10  *RP_H_* ～*U*(1.2,1.3), p=0.00 | *RP_F_* ～*U*(0.2,0.4), p=0.40  *RP_F_* ～*U*(0.4,0.6), p=0.30  *RP_F_* ～*U*(0.6,0.8), p=0.14  *RP_F_* ～*U*(0.8,1.0), p=0.06  *RP_F_* ～*U*(1.0,1.2), p=0.08  *RP_F_* ～*U*(1.2,1.3), p=0.02 |
|  | Radius reduction factor | *rP_Z_* ～*U*（-0.003，-0.001） | *rP_H_* ～*U*（-0.003，-0.001） | *rP_F_* ～*U*（-0.003，-0.001） |
|  | Axial deviation Angle（°） | *aP_Z_* ～*N*（0.40，0.18^2^） | *aP_H_* ～*N*（0.45，0.20^2^） | *aP_F_* ～*N*（0.56，0.25^2^） |
|  | Radial deflection Angle（°） | *jP_Z_* ～*U*(0,0.04), p=0.26  *jP_Z_*～*U*(0.04,0.08), p=0.40  *jP_Z_*～*U*(0.08,0.12), p=0.20  *jP_Z_*～*U*(0.12,0.16), p=0.08  *jP_Z_*～*U*(0.16,0.20), p=0.02  *jP_Z_* ～*U*(0.20,0.24), p=0.04 | *jP_H_* ～*U*(0.02,0.05), p=0.20  *jP_H_*～*U*(0.05,0.08), p=0.28  *jP_H_*～*U*(0.08,0.11), p=0.32  *jP_H_*～*U*(0.11,0.14), p=0.16  *jP_H_*～*U*(0.14,0.17), p=0.00  *jP_H_* ～*U*(0.17,0.20), p=0.04 | *jP_F_* ～*U*(0.02,0.05), p=0.24  *jP_F_*～*U*(0.05,0.08), p=0.22  *jP_F_*～*U*(0.08,0.11), p=0.22  *jP_F_*～*U*(0.11,0.14), p=0.20  *jP_F_*～*U*(0.14,0.17), p=0.06  *jP_F_* ～*U*(0.17,0.20), p=0.06 |
|  | Number | *NP_Z_* ～*U*（9，12） | *NP_H_* ～*U*（11，14） | *NP_F_* ～*U*（12，15） |
| **Tuber** | Potato shape | long potato， p=0.09  ellipsoid potato，p=0.72  spherical potato，p=0.19 | long potato， p=0.24  ellipsoid potato，p=0.75  spherical potato，p=0.01 | long potato， p=0.00  ellipsoid potato，p=0.77  spherical potato，p=0.23 |
|  | Size in the longest axis (mm) | *LK_Z_* ～*U*(40,55), p=0.14  *LK_Z_* ～*U*(55,70), p=0.34  *LK_Z_* ～*U*(70,85), p=0.12  *LK_Z_* ～*U*(85,100), p=0.20  *LK_Z_* ～*U*(100,1115), p=0.10  *LK_Z_* ～*U*(115,130), p=0.10 | *LK_H_* ～*U*(40,60), p=0.23  *LK_H_* ～*U*(60,80), p=0.33  *LK_H_* ～*U*(80,100), p=0.16  *LK_H_* ～*U*(100,120), p=0.18  *LK_H_* ～*U*(120,140), p=0.04  *LK_H_* ～*U*(140,150), p=0.06 | *LK_F_* ～*U*(40,50), p=0.22  *LK_F_* ～*U*(50,60), p=0.16  *LK_F_* ～*U*(60,70), p=0.30  *LK_F_* ～*U*(70,80), p=0.14  *LK_F_* ～*U*(80,90), p=0.14  *LK_F_* ～*U*(90,100), p=0.04 |

Note: p means probability.

“X～*U*(a,b), p=c ” means the random variable *X* follows the uniform distribution on the interval [a, b], and the probability is c ; such as “*LK*_Z_ ～*U*(40,55), p=0.14” means that the longest axis size of the Zaodabai tuber follows the uniform distribution on interval [40, 55] , and the probability is 0.14.

*“X*～*U*(a,b) ” means the random variable *X* follows the uniform distribution on the interval [a, b].

“*X*～*N*(a,b^2^)” means the random variable *X* has a normal distribution, where a is the mean and b is the standard deviation.
